# Tracking solutions to a persistent threat: spatial movement patterns reflect lead exposure in critically endangered California condors

**DOI:** 10.1007/s10646-025-02921-9

**Published:** 2025-07-05

**Authors:** Varalika Jain, Christopher J.W. McClure, Chris N. Parish, Timothy J. Hauck, Petra Sumasgutner

**Affiliations:** 1 Konrad Lorenz Research Center for Behavior and Cognition, Core facility of the University of Vienna, Grünau im Almtal, Austria; 2 Department of Behavioral and Cognitive Biology, University of Vienna, Vienna, Austria; 3 The Peregrine Fund, Boise, ID USA

**Keywords:** Animal movement, Conservation, Detection, Endangered species, Lead poisoning, Toxic ammunition

## Abstract

Lead contamination, an exclusively human-induced issue, poses a serious threat to the critically endangered California Condor (*Gymnogyps californianus*). Contamination occurs through condors’ ingestion of lead ammunition residues embedded within the remains of shot animals. Detecting lead exposures typically requires resource-intensive and invasive interventions. Here, we explored a minimally invasive alternative, analyzing movement data from GPS-tagged condors in the 30-day period prior to when their blood lead levels were measured. We found spatial, but not temporal, differences in movement patterns. Lead-exposed condors traversed larger areas of the landscape, with ranges more concentrated in a previously identified high-risk zone, and shared space more extensively with one another than unexposed individuals. However, lead-exposed condors did not exhibit declining movement patterns through time when compared to unexposed birds, making movement-inferred post-exposure detection difficult. While GPS-telemetry is a useful tool in understanding condors’ spatial patterns in relation to lead exposure, future research exploring how movement patterns can be used to detect, predict, and provide early warnings of at-risk condors may better inform targeted conservation efforts.

## Introduction

Avian scavengers belong to the most imperiled functional guild and face persistent threats from anthropogenic activities (Buechley and Şekercioğlu 2016). Through carrion consumption, avian scavengers contribute invaluably to disease control and nutrient recycling, playing a vital role in maintaining ecosystem health (Sekercioglu 2006, Markandya et al. 2008). However, in anthropogenic landscapes these ecosystem services often come at the cost of exposure to harmful and potentially deadly contaminants, including pesticides, medical drugs, and lead (Pain et al. 2009, Ogada et al. 2012, Jimenez-Lopez et al. 2021, Plaza et al. 2022). Anthropogenic toxic agents are the main cause of mortality and illness in obligate avian scavengers – vultures and condors – with lead being the primary contributor (Buechley and Şekercioğlu 2016, Plaza and Lambertucci 2019, Ives et al. 2022). Lead contamination is cautioned as a pervasive, silent threat due to its often-undetectable infiltration into the environment and food web. While a single, severe poisoning event can be life-threatening (Pikula et al. 2013), repeated exposures to lead can also result in it gradually accumulating in an individual over time (Parish et al. 2007, Krone 2018). Throughout an individual’s lifetime, lead exposures can impact reproductive, metabolic, immune, nervous, and muscular system functioning, as well as behavior – causing significant harm (Gangoso et al. 2009, Vallverdú-Coll et al. 2019, van den Heever et al. 2019). Protecting avian scavengers, and their roles in maintaining ecological balance, requires timely monitoring, detection, and treatment of lead-exposed individuals (Ogada et al. 2012, Buechley and Şekercioğlu 2016, Pain et al. 2019).

For the critically endangered California Condor (*Gymnogyps californianus*), lead poisoning has been a persistent threat for decades (Cade 2007, Parish et al. 2009, Finkelstein et al. 2012). In the 1980s, lead contamination arising from anthropogenic hunting activity was identified as a prominent concern and contributor of rapid condor population declines (Snyder and Snyder 1989). Population numbers had dropped so low that by 1987, the last free-roaming condors were all in captivity – forming a total population of only 27 captive individuals (Meretsky et al. 2000, Church et al. 2006, Cade 2007). Since then, intensive captive-management and continual releases of captive-reared birds from facilities in California, Baja California, and Arizona have given rise to over 300 free-flying individuals to date (Hunt et al. 2009, Sieg et al. 2009). As a species with low reproductive potential and delayed maturity, rearing in captivity improves both population numbers and survival rates, relative to the increased causes of morbidity and mortality arising from anthropogenic sources (i.e., preventable lead poisoning) (Cade 2007). Alongside captive-releases, a variety of regulatory efforts targeting lead-based hunting approaches have been implemented since 1996 (Schulz et al. 2023). Despite these conservation efforts, the persistence of lead exposure remains the most significant hurdle preventing recovery in the California Condor and is the leading cause of death across its range (Meretsky et al. 2000, Mee and Snyder 2007, Hunt et al. 2009, Finkelstein et al. 2012).

The primary lead exposure pathway is through the ingestion of lead fragments, pellets, and sometimes whole bullets from ammunition, which remain in carrion and gut piles of shot animals (Pain et al. 2009, Plaza and Lambertucci 2019, Ives et al. 2022). Radiographs of carcasses have shown that a single bullet can shatter into over 200 fragments (Hunt et al. 2006). Because condors feed communally, a single contaminated carcass is capable of poisoning multiple individuals (Woods et al. 2007, Ogada et al. 2012, Plaza and Lambertucci 2019). Condors forage widely, flying far to exploit transient food sources across the landscape (Hall et al. 2019, Hall et al. 2021). For individuals released from captivity, these movements reflect behavior typical of wild condors (Rivers et al. 2014, Bakker et al. 2017). Captive-reared condors, released as juveniles, are initially largely dependent on supplementary food provisions (such as dairy calves) near managed release sites(Kelly et al. 2014). Increasing in age and independence from intensive management often involves a shift from smaller movements near managed release sites to larger movements across the landscape, and thus a reduced reliance on lead-free food to an increased consumption of ephemeral offal and carrion that may contain lead (Hunt et al. 2006, Parish et al. 2009, Bakker et al. 2017). Fortunately, recaptures and health evaluations have been possible because individuals, despite roaming extensively, sometimes return to known supplementary feeding stations – a valuable management opportunity (Cade 2007).

The silent nature of lead contamination means that it can go unnoticed until irreversible damage is already done, underscoring the importance of regular testing and vigilant prevention measures to ensure condor survival. Detecting lead exposures is a complex task involving invasive and resource-intensive blood sampling methods (Church et al. 2006, Parish et al. 2007). However, less invasive approaches such as inferences based on behavioral analyses from GPS-telemetry data could offer valuable alternative avenues. For instance, behaviors symptomatic of lead poisoning can involve neurological issues and muscle weakness that can impair movement, resulting in a reduced ability to fly [(Fry and Maurer 2003, Wynne and Stringfield 2007, Pikula et al. 2013, Ecke et al. 2017) but see (Poessel et al. 2018)]. Here, we combine data from routine field monitoring of lead contamination with GPS-telemetry data to evaluate whether movement patterns and ranging behavior differ between exposed and unexposed condors. Given the toxic effects of lead, we first predicted that lead-exposed birds will show a decline in their movement patterns as they approach the blood lead-level testing date, reflecting deteriorating health from lead poisoning (Fry and Maurer 2003), while birds with background lead levels (i.e., no traces of recent exposure) will maintain consistent movement patterns. Patterns of movement declines in lead-exposed could aid in the development of an exposure-detection system. Second, we predicted that in the 30-days prior to being tested for lead, birds tested for higher levels of lead will have larger movement ranges compared to birds with only background levels of lead. This is based on literature that has shown birds with an increased lead exposure risk to also exhibit larger ranging behavior and a decreased reliance on lead-free supplementary food (Kelly et al. 2014, Rivers et al. 2014, Bakker et al. 2017). Third, we examined how condors of different lead exposure levels use and share space across the landscape – specifically in relation to a previously identified high-risk zone – and with each other. We assumed condors with background levels of lead to have smaller ranges and to be feeding communally on managed lead-free supplementary food. Thus, we expected them to overlap more with one another than with lead-exposed individuals. We also expected lead-exposed individuals to overlap more with each other than with background level birds, assuming multiple individuals feed on the same contaminated carcass across the landscape.

## Methods

### Study site and system

Since the first release in 1996 by The Peregrine Fund, more than 250 individually marked condors have been released in northern Arizona (approximately 36° N latitude, 112° W longitude) to support species recovery efforts and establish a self-sustaining population in the wild. The northern Arizona population comprises 26% of total free-flying individuals. Supported by the rugged terrain, comprising canyons and plateaus, with diverse habitats including desert scrub, semi-arid grasslands, and woodlands, and elevation ranging from 600–2800 meters above sea level, the condor population currently ranges across both northern Arizona and southern Utah (Parish et al. 2007). Cliffs, winds, and elevated summer temperatures create energetically beneficial updraft wind conditions that enable the condors to traverse this expansive landscape. In this region, condors typically feed on carrion of ungulate species (Hunt et al. 2007), but numerous cases of smaller animals have been documented – including coyotes (*Canis latrans*). During hunting season in autumn, these sources of carrion or gut piles pose a threat to the condors, particularly in the Kaibab Plateau and North zones, because ungulates are traditionally shot with lead-based bullets. Lead toxicity from ingestion is the leading cause of mortality in this population and threatens its self-sustainability (Hunt et al. 2006, Parish et al. 2007, Woods et al. 2007, Green et al. 2008).

### Testing and treatment

Hundreds of condors in northern Arizona have been recaptured for lead exposure testing and diagnosis since 1996 (Hunt et al. 2007, Parish et al. 2007, Sieg et al. 2009, Pikula et al. 2013). Trapped free ranging condors are tested for lead exposure throughout the year, with efforts concentrated during the autumn hunting season, when condors are more likely to encounter lead from spent bullet fragments in carrion (Hunt et al. 2006, Hunt et al. 2007). As described in Parish et al. (2007), condors are caught using a “walk-in” chain trap (3.7 m × 3.7 m × 1.6 m). Some individuals are also target-captured for testing in rare instances where severe lead contamination events result in immobility, preventing a bird from returning to the capture site. A sample of approximately 1–3 ml of blood is taken from the medial-tarsal vein using a 22-gauge needle, using standard blood collection techniques (Espín et al. 2021). Samples are stored in heparinized tubes. For lead contamination analysis, 50 µl of whole blood from each sample is transferred to a vial containing 250 µl of 0.35 molar HCl. This is then placed on a sensor strip inserted into a portable lead analyzer (LeadCare® Blood Lead Testing System, ESA Inc, Chelmsford, MA, USA) (Fry and Maurer 2003). This instrument measures and displays lead values between 0–65 µg/dl, but studies have revealed that these instruments under-report values compared to laboratory assays (Parish et al. 2007, Finkelstein et al. 2014, Herring et al. 2018).

‘Background’ lead levels are considered between 0–14 µg/dl (Fry and Maurer 2003, Parish et al. 2007). ‘Exposures’ are defined by blood lead concentrations between 15–29 µg/dl and ‘high exposures’ by concentrations between 31–59 µg/dl. Above 60 µg/dl, birds are classified as being ‘clinically affected’ (Fry and Maurer 2003, Cade 2007). Individuals with high lead levels (~60 µg/dl) are almost always taken into captivity. They are started on chelation therapy depending on whether lead levels continue to increase in subsequent days, or if levels are continuously high over time (Parish et al. 2007). Chelation therapy consists of intramuscular injections of Ca EDTA (calcium edetate) twice daily for 5 days – a procedure that usually produces a rapid decrease in blood lead levels (Hunt et al. 2009).

### GPS data

Birds were equipped with Microwave Telemetry, Inc. Solar Argos/GPS 50 g Patagial PTTs (https://www.microwavetelemetry.com/solar_argos_gps_50g_patagial_ptt) from 2015 with locations sampled at hourly intervals. We sourced data from 52 GPS-tagged California Condors (males = 28, females = 24) from 14th of January 2015 till the 29th of January 2023. We first cleaned the data for any outliers based on spatial limits (longitude range: −120–−90, latitude range: 30–39.5), angle, and speed, removing points of both high speed (incoming or outgoing ≥ 0.8 ms^−1^) and large turning angles (≥150°) (Figure S1) (Gupte et al. 2022). For individuals whose daily tracks had missing points at the scheduled hourly intervals, we interpolated the track (Kranstauber et al. 2020). We also specified and filtered daytime GPS fixes using the dawn and sunset times specific to the latitude and longitude of each GPS fix, given its associated timestamp.

For each individual, we filtered tracking data to 30 days prior to the date of testing (i.e., approximately two half-lives of ~13 days of lead in condor blood (Fry and Maurer 2003)). Because some individuals were tested repeatedly within a short period of time, we only included consecutive tests conducted at least 30 days apart. We also ensured that no birds underwent clinical care or treatment in the 30 days prior to when they were tested for lead poisoning. We considered individuals with *n* > 3 fixes per day, out of a maximum of 14 possible fixes per day (Figures S2 and S3). We had a total of 43 individual-test instances for ‘Background’ level birds, 53 for ‘Exposure’ level birds, 29 for ‘High Exposure’ level birds, and 31 for ‘Clinically affected’ birds.

To test our first hypothesis that lead-exposed birds experience deteriorating health and thus movement patterns from lead poisoning, we looked at four **daily movement metrics:** (1) **average hourly step length per day**, which reflects the distance (in km) covered between two consecutively recorded points; (2) **average net squared displacement (NSD) per day**, which captures the squared distance (km^2^) of each point from the first location of the track; (3) **daily mean squared displacement (MSD)**, representing the average squared distance (km^2^) between each point and the mean point; and, (4) **daily minimum convex polygon (MCP)** per individual, capturing the extent of the distribution of locations (km^2^).

**Fig. 1:**
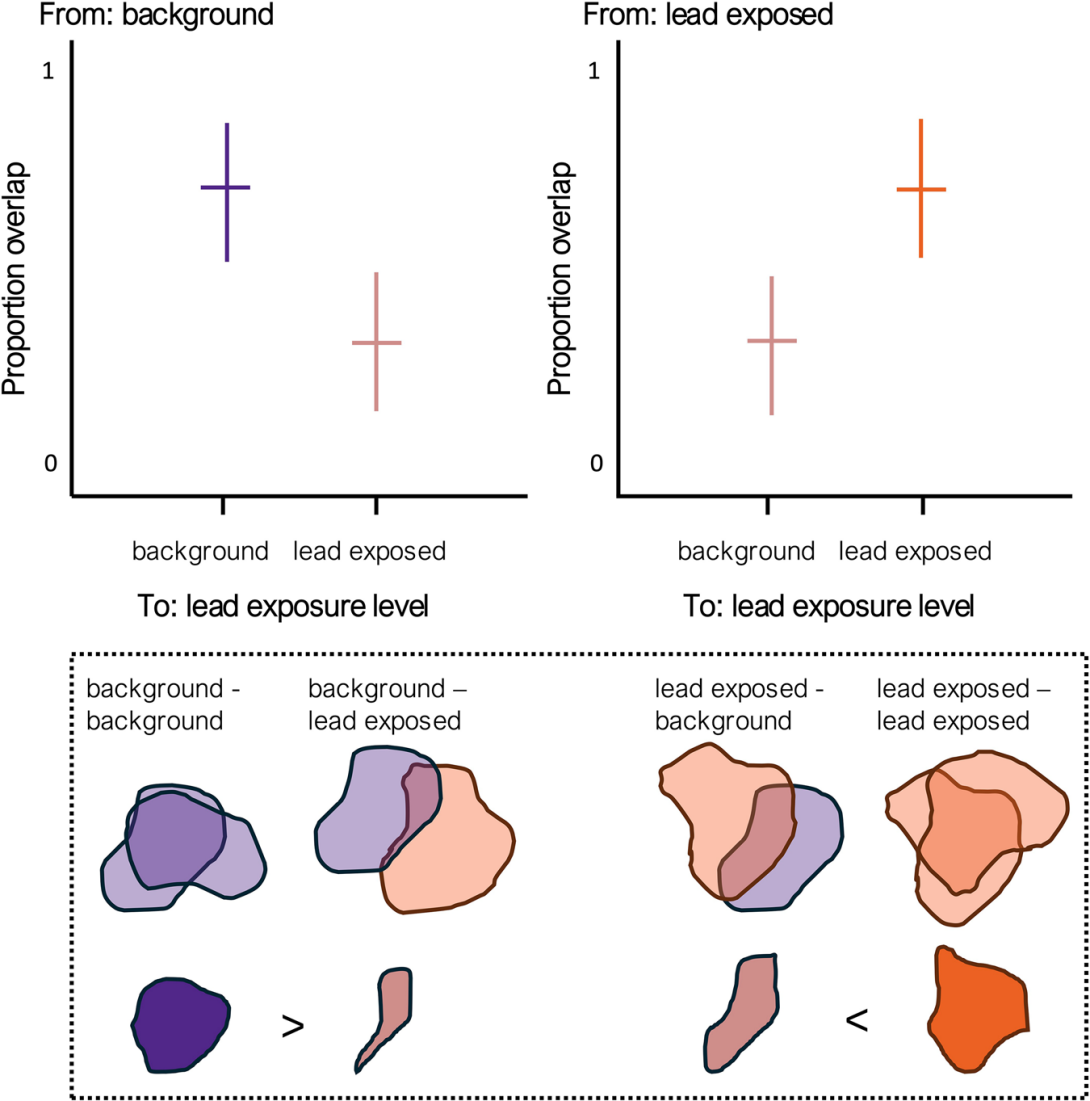
A conceptual image representing the directional range overlap among autocorrelated Kernel Density Estimates (aKDEs). We expect Californian condors *(Gymnogyps californianus)* with background lead levels to show greater overlap in their range use compared to lead-exposed individuals. Conversely, we expect lead-exposed individuals to overlap more with each other than with background level birds

To test our second and third hypotheses regarding ranging behavior and space-use differences among birds of different lead exposure levels, we first computed the **autocorrelated Kernel Density Estimate (aKDE)** by fitting a continuous time movement model to the data at the 95% isopleth level (Signer et al. 2019, Signer and Fieberg 2021). We used the aKDE method because an individual’s positions along its track is often spatially and temporarily not independent, and thus a specific point in time can be correlated to its past and future positions (i.e., autocorrelated) (Fleming et al. 2015, Silva et al. 2022). We also used the aKDEs to assess ranges in relation to a previously identified high-risk zone – hunting unit 12 A in Arizona, covering a large part of the Kaibab Plateau (36.5832° N, 112.1676° W). For each aKDE, we did so by measuring the ratio of the area where the range intersected with the hunting unit to the total aKDE area. Using the aKDEs, we then measured pairwise combinations of the **directional proportion of range overlap** (i.e., range_*i,j*_ ≠ range_*j,i*_) across the 30-day period prior to testing (Fig. 1).

We conducted all GPS data processing in R (version 4.3.3, (R Core Team 2024)). We removed outliers following the ‘atlastools’ package guide (Gupte et al. 2022). Interpolation of gappy hourly intervals in tracks was done using the ‘move’ package (Kranstauber et al. 2020). The ‘suncalc’ package was used to filter daytime datapoints (Thieurmel and Elmarhraoui 2019). All movement metrics, the aKDEs, and overlaps were calculated using the ‘amt’ package (Signer et al. 2019).

### Statistical analysis

For each of the four daily movement metric responses, we used linear mixed models with the fixed effects of lead level, day prior to testing, and their interaction. We included the interaction term given our assumption that lead-exposed individuals move widely across the landscape and likely encounter contaminated carrion, and our expectation that they will experience reduced movements closer to when they were detected with poisoning due to known negative health impacts on movement capacities. As lead can accumulate over an individual’s lifetime and as older individuals tend to be more exposed to lead (Hall et al. 2007, Kelly et al. 2014), we controlled for the age of the individual as a fixed effect in the model. We also included the random intercepts of the individual identity, and the testing date nested in individuals (i.e., we controlled for individuals that were tested for lead poisoning multiple times in the dataset). All theoretically identifiable random slopes in the model were included to avoid overconfident model estimates and to keep type I error rates at the nominal level of 5% (Schielzeth and Forstmeier 2009, Barr et al. 2013). Specifically, we included the random slopes of day prior, lead level, their interaction, and age within individual. We also included day prior and age within the testing date nested in individuals. Lead level was manually dummy coded and centered to a mean of zero (Kraemer and Blasey 2004, Alkharusi 2012, Cohen et al. 2013).

To estimate the amount of range overlap between individuals of different lead levels, we fitted a Generalized Linear Mixed Model (GLMM) with a beta error distribution and logit link function (Bolker 2008, Bolker et al. 2009). The lead level combination (e.g., from background – to background, from background - to exposure) was the only fixed effect in this model. We included the random intercept of the individual-identity dyad, and the random slopes of lead level combination within the intercept. Lead level combination was manually dummy coded and centered to a mean of zero.

We began with fitting maximal models for all the responses, where we included the parameters for the correlations among random slopes and intercepts (Barr et al. 2013). However, because this resulted in estimated correlations being unidentifiable or with the model failing to converge, we excluded them from all models except that of the MCP response (Matuschek et al. 2017). This resulted in only a minor reduction in the model fits (log-likelihoods of models with all correlation parameters included, and without: step length *−8038.766 [df* = *61], −8046.991 [df* = *22]*; NSD *−11 711.95 [df* = *61], −11 718.22 [df* = *22]*; MSD *−11 668.34 [df* = *61], −11 674.45 [df* = *22]*; overlap model failed to converge).

As an overall test of the fixed effects, we conducted a full-null model comparison aimed at avoiding cryptic multiple testing (Forstmeier and Schielzeth 2011). In the daily movement models, we tested for the effect of lead level, day prior, and their interaction. The resulting null model comprised of only our control variable, age. In the overlap model, we tested for the effect of the lead level combination and our null model comprised of only the intercept. The null models had the same random effect and slope structure as the full model. The comparison was based on a likelihood ratio test (Dobson and Barnett 2018) and only if this revealed significance, we further investigated the influence of individual predictor variables.

Prior to fitting each model in R (version 4.3.3, (R Core Team 2024)), we inspected the distribution of predictors and the response for symmetry and outliers. In the daily movement metric models, we z-transformed the covariates day prior and age to achieve an easier interpretability (Schielzeth 2010) and to ease model convergence. Regarding the response, because all four daily movement metrics included true zero values, we investigated transforming the response by applying either a ‘log plus one’ transformation or by adding a constant (i.e., half the smallest non-zero value) to it and then applying a log-transformation. We found that the first method provided a visually normally distributed response for the step length, NSD, and MSD, and that the second method provided a visually normally distributed response for the MCP. Additionally, in all daily movement metric models, we gave more weight to the response supported by more GPS fix locations. For the overlap model, we transformed the response to avoid values being exactly zero or one (Smithson and Verkuilen 2006).

We fitted the model for the daily movement metrics using the ‘lmer’ function of ‘lme4’ (Bates et al. 2015) and the model for the proportion overlap in aKDE using the ‘glmmTMB’ function from ‘glmmTMB’ (Brooks et al. 2017). For all daily movement metric models, we checked whether the assumptions of normally distributed and homogenous residuals were fulfilled by visual inspection of Q-Q plot of residuals against fitted values (Field 2005). We did not find any overdispersion for the overlap model (dispersion parameter: 0.856). The daily movement metric models contained more than one predictor and so we tested for collinearity issues by specifying a model without the interaction. Using Variance Inflation Factors (VIF), we found no assumptions violated (all VIF < 2) (Quinn and Keough 2002, O’Brien 2007). We determined model stability on the level of the estimated coefficients and standard deviations by excluding the levels of the grouping factors one at a time (Nieuwenhuis et al. 2012). All models were of robust stability (see minimum and maximum of model estimates in Tables S1 and S5). For models that revealed a significant full-null model comparison, we obtained confidence intervals of model estimates and fitted values using a parametric bootstrap. Lastly, we report the significance of individual fixed effects by means of likelihood ratio tests (Dobson and Barnett 2018) comparing the full model with models lacking the terms of interest one at a time (R function ‘drop1’).

The daily movement metric models comprised of 4362 observations of 52 individuals and 156 individual-test combinations. For the step length, NSD, and MSD we had approximately 198 observations per estimated term. The MCP model had approximately 364 observations per estimated term. The overlap model comprised of 32 580 directional overlap proportions from 1693 individual-identity dyads, with 5430 observations per estimated term.

## Results

In the 30 days prior to when they were tested for lead poisoning, the condors’ averages for the daily metrics were 2.79 km in mean hourly step length, 282.44 km^2^ in mean Net Squared Displacement (NSD), 141.76 km^2^ in Mean Squared Displacement (MSD), and 78.30 km^2^ in Minimum Convex Polygon (MCP). The autocorrelated Kernel Density Estimates (aKDEs) revealed that their range sizes over this period were on average 8321.04 km^2^.

**Fig. 2:**
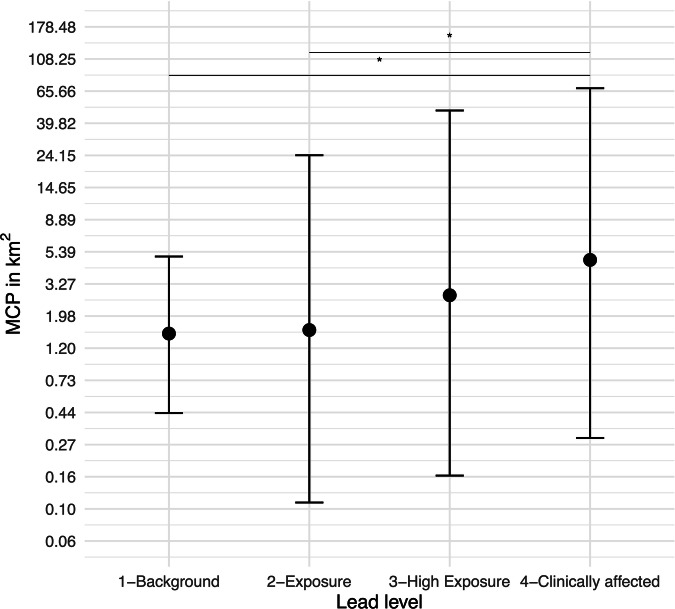
Minimum Convex Polygons (MCPs) of California Condors *(Gymnogyps californianus)* with different lead exposure levels. Clinically affected birds had notably larger daily distributions compared to background and exposed level birds, as indicated by the asterisk (*). The crosses represent the fitted model and its confidence limits (conditional on all covariates and factors centered to a mean of zero). Confidence limits were obtained through bootstrapping the data 1000 times and determining the 95% confidence interval. Full model details in Table S4

Overall, neither lead exposure level, day prior to testing, nor their interaction influenced the step length (full-null model comparison: χ^2^ = 6.56, df = 7, *p* = 0.472), NSD (full-null model comparison: χ^2^ = 8.39, df = 7, *p* = 0.299), nor MSD (full-null model comparison: χ^2^ = 8.63, df = 7, *p* = 0.280). The full-null model comparison for the MCP was significant (χ^2^ = 19.38, df = 7, *p* = 0.007), but the interaction between lead exposure level and day prior did not reveal significance (χ^2^ = 1.46, df = 3, *p* = 0.693) (Tables S1 and S2). After removal of the non-significant interaction, we found that lead level influenced MCP (Table S3), with post-hoc comparisons revealing clinically affected birds having notably larger daily distribution of points than background and exposure level birds (Fig. 2, Table S4). We observed a slight decreasing trend in MCP sizes closer to the date of blood-lead level testing and found that MCP sizes tended to increase with the age of the birds (Tables S1 and S3).

**Fig. 3:**
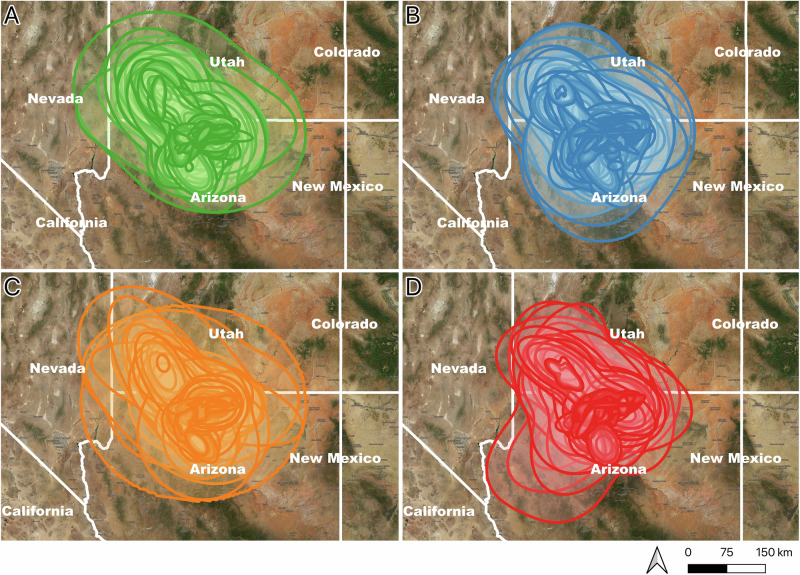
The thirty-day Autocorrelated Kernel Density Estimates (aKDEs) for California Condors *(Gymnogyps californianus)* with different lead exposure levels revealed **A** background level birds had the smallest average aKDEs (mean: 6907.72, range: 0.91–48266.56 km^2^), followed by **B** exposure level birds (mean: 7721.90, range: 208.96–43414.52 km^2^), then **C** high exposure birds(mean: 9472.57, range: 0.00–40961.33 km^2^), and lastly **D** clinically affected birds (mean: 10317.93, range: 350.37–51079.10 km^2^)

There was wide variation in the aKDE values per lead exposure level. This value was highest on average for clinically affected individuals (mean: 10 317.93, range: 350.37–51 079.10 km^2^), followed by high exposure birds (mean: 9472.57, range: 0.00–40 961.33 km^2^), exposure level birds (mean: 7721.90, range: 208.96–43 414.52 km^2^), and lastly background level birds (mean: 6907.72, range: 0.91–48 266.56 km^2^) (Fig. 3).

**Fig. 4:**
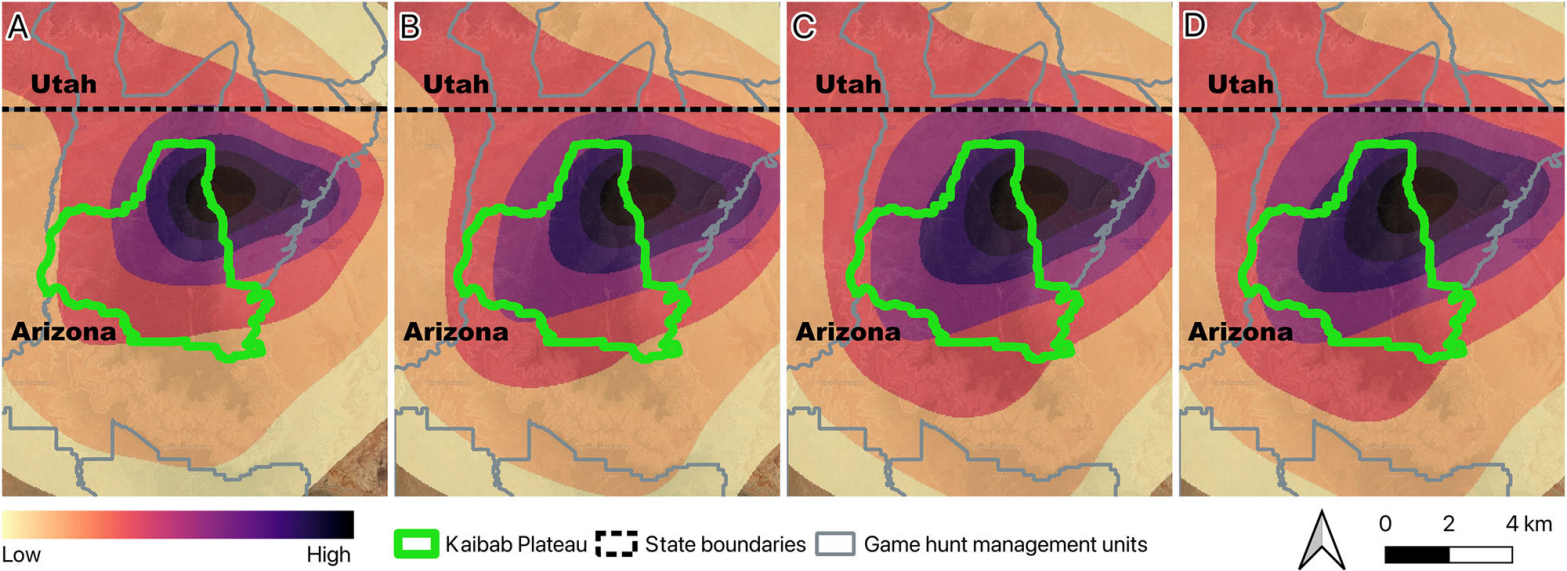
Heatmaps depicting the concentration of autocorrelated Kernel Density Estimates (aKDEs) for California Condors *(Gymnogyps californianus)* intersecting with the Kaibab Plateau zone (outlined in green) - a previously identified risk area for lead exposures. The ratio of the area where the aKDE intersects with the Kaibab Plateau to the total aKDE area was on average **A** 0.29 for background, **B** 0.28 for exposure, **C** 0.27 for high exposure, and **D** 0.36 for clinically affected level birds. Increasing in concentration of ranges around the Kaibab Plateau zone are indicated by darker colors

We found that birds of increasing lead exposure level appeared to have more of their ranges concentrated in the hunting unit 12 A than background level birds (Fig. 4). The ratio of the area where the aKDE intersected with the hunting unit to the total aKDE area was on average 0.29 for background, 0.28 for exposure, 0.27 for high exposure, and 0.36 for clinically affected level birds.

**Fig. 5:**
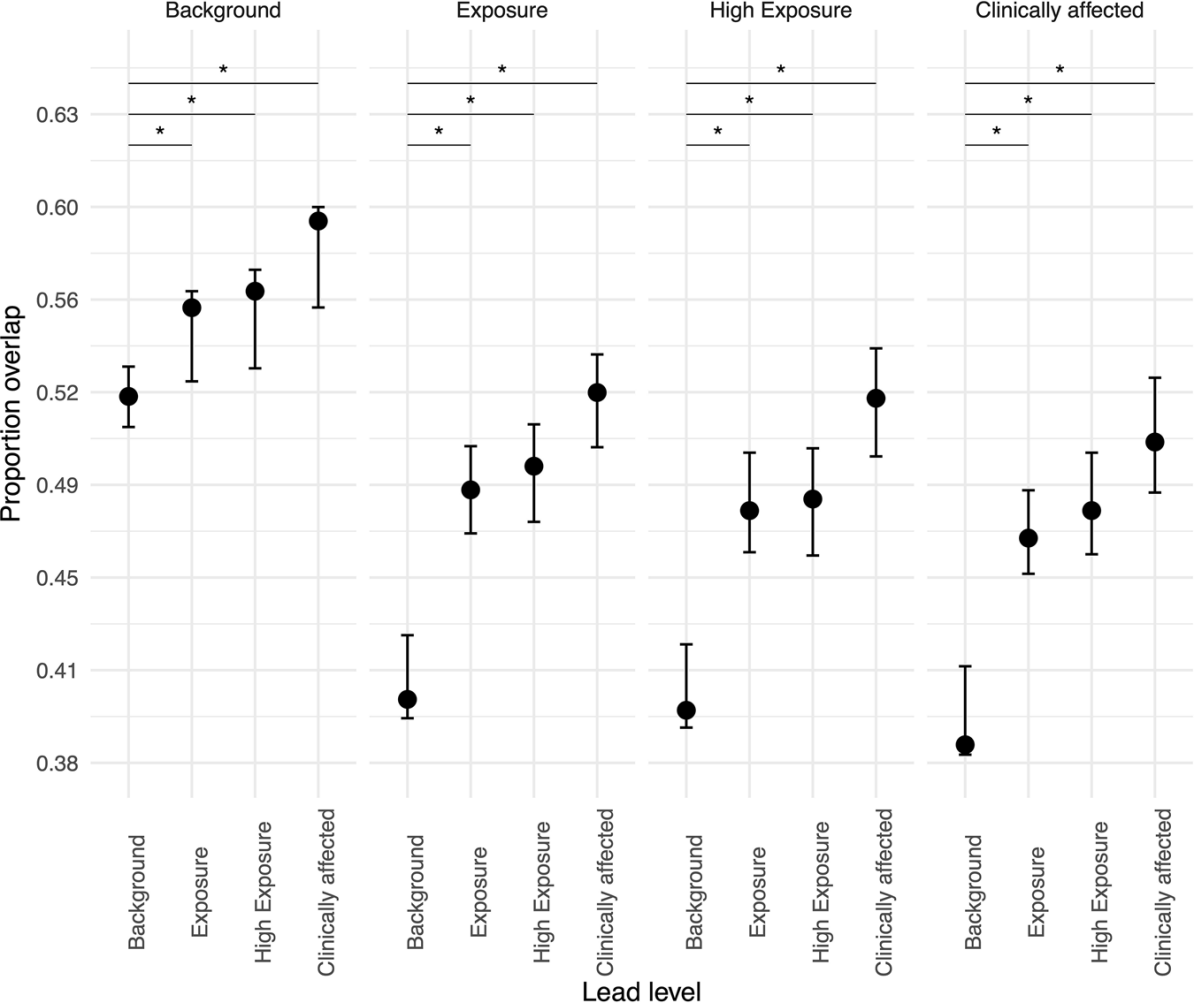
Proportion spatial overlap for California Condors *(Gymnogyps californianus)* with different lead exposure levels. The crosses represent the fitted model and its confidence limits (conditional on all covariates and factors centered to a mean of zero). Confidence limits were obtained through bootstrapping the data 100 times and determining the 95% confidence interval. Across all four lead levels overall, birds had the lowest proportion of overlap with background level birds and the most with clinically affected birds. Overlap with background-level birds were notably lower than overlap with non-background level birds. Pairwise comparisons only from the background-level to non-background levels are shown (depicted by ‘*’, Table S7)

We found an influence of lead exposure level combinations on the proportion of spatial overlap (full-null model comparison: χ^2^ = 613.86, df = 15, *p* = < 0.001) (Fig. 5, Tables S5 and S6). Across all four lead levels overall, birds had the lowest proportion of overlap with background level birds and the most with clinically affected birds. Overlap with background-level birds were notably lower than overlap with non-background level birds (Table S7).

## Discussion

We explored the potential of using satellite-based GPS telemetry to detect lead exposures in the California Condors by analyzing their movement behavior. Our study on condors in northern Arizona and southern Utah shows that condors with higher lead levels exhibited different spatial movement patterns compared to those with lower exposure, however this did not vary temporally. These distinct spatial pattern findings, yet lack of temporal trends, provide important insights into the difficulties of detecting lead-exposed individuals. Based on our findings, we propose opportunities for future research to aid the conservation management of condors and other avian scavengers against a persistent anthropogenic threat.

Among avian scavengers, lead toxicity can lead to a variety of sublethal symptoms (Golden et al. 2016). In condors, this affects their nervous and muscular systems, potentially causing neurological issues and muscle weakness that can impair movement (Wynne and Stringfield 2007, Walters et al. 2010). However, contrary to our first prediction, we found no evidence for movement impairment in the metrics we assessed – lead-exposed birds did not show declining trends, compared to non-exposed birds, in the 30 days leading up to when they were tested for lead. The lack of observed movement response to lead highlights the multifaceted challenges posed by the birds’ behavioral responses, nature of lead exposure, and the limitations of current methodologies, with potential implications for other avian scavengers.

Raptors, including condors, can exhibit a wide range of clinical symptoms to lead exposure that develop at different rates – from a few days, to up to 20 weeks – making the time interval between exposure, onset of clinical symptoms, and death highly variable (Pattee et al. 1981, Fry and Maurer 2003, Pattee et al. 2006, Golden et al. 2016). In condors, birds with > 60 µg/dl blood lead concentration are regarded as clinically affected and often display clinical symptoms and birds with > 100 µg/dl blood lead concentration experience acute toxicity (Fry and Maurer 2003). Instead of a gradually declining response to lead exposure, birds might only show a sharp drop in movement (i.e., immobility) once a critical level of toxicity is exceeded and symptoms become debilitating (Fry and Maurer 2003, Poessel et al. 2018). In such a scenario, only severe lead contamination might be detectable though GPS data, appearing as a sudden and potentially prolonged period of no flight activity (Krone et al. 2009, Duriez et al. 2023). As soarers, condors and other large-bodied raptors rely on low-energy flight that allows them to traverse vast landscapes even when unwell, especially under optimal weather conditions (Duriez et al. 2014, Williams et al. 2020). With movements of such magnitude, a bird exposed to lead could probably still fly far distances before experiencing a state of symptom-severity that immobilizes it.

Lead exposure events, arising from the ingestion of spent ammunition residues in carrion, are challenging to identify both spatially and temporally. Condors exploit carrion that are both spatially unpredictable and temporally ephemeral (Poessel et al. 2018), making it difficult to pinpoint when and where they fed on a contaminated carcass (Pain et al. 2009, Plaza and Lambertucci 2019, Ives et al. 2022). Field blood tests provide only a snapshot of lead concentration levels, representing approximately 10% of a condor’s annual exposure history (Finkelstein et al. 2012, Bakker et al. 2017, Poessel et al. 2018). As a result, these tests are unlikely to capture the peak magnitude or frequency of lead exposure events. Furthermore, repeated lead exposures and medical treatments can alter how individuals respond to subsequent exposures. For instance, birds with a history of acute poisoning may react more sensitively to lower levels of lead than birds with no prior exposure (Poessel et al. 2018). Rast et al. (2024) demonstrated how combining both GPS-inferred spatial clusters and accelerometer patterns from African white-backed vultures (*Gyps africanus*) could be used to successfully detect carcasses across the landscape. While this could serve as an opportunity for detecting exposure events in condors, it would also require extensive groundwork to validate, in real time, the presence or absence of a carcasses arising from clusters.

Movement characteristics based on data collected at hourly intervals are limited to inferences made at that temporal resolution (Nathan et al. 2022), and may be too coarse to detect subtler movement impairments caused by lead exposure (Ecke et al. 2017, Poessel et al. 2018). For instance, a GPS-fix at an hourly resolution can show whether an individual is still moving across the landscape, likely using non-energetically demanding soaring flight. However, it misses out on the finer-scaled measurements, such as recording the demanding effort required to take-off from the ground (i.e., engaging in flapping flight and sustaining a higher heart rate), which lead-poisoned individuals might struggle to do more than healthy individuals (Duriez et al. 2014, Williams et al. 2020). To detect changes in finer-scale movements, high-resolution accelerometer data could be explored, offering insights into detailed behavioral patterns, (Nathan et al. 2012), energy expenditure (Wilson et al. 2020), and activity levels (Krone et al. 2009). Such an application of using multiple fine-scale sensors was demonstrated in the investigation of an anthropogenic toxin-induced mortality of recently fledged cinereous vulture (*Aegypius monachus*) (Herrero-Villar et al. 2021).

Our findings pertaining to our second prediction – lead exposed birds exhibit larger ranges – were supported in both daily ranging (MCP) behavior trends and the overall 30-day ranges (aKDE). This aligns with existing studies on condor range sizes (Kelly et al. 2014, Rivers et al. 2014, Bakker et al. 2017), suggesting that when condors exhibit larger ranging behavior, they depend less on concentrated supplementary feeding sites and more on carrion scattered across the landscape. The tendency to travel vast distances reflects increasingly wild type behaviors, which is positive and desirable for a self-sustaining population that originates from captive-bred individuals (Cade 2007, Kelly et al. 2014). However, this behavior unfortunately also increases the risk of encountering lead-contaminated food sources. Our results also support a trend that ranging behavior increases with age (Kelly et al. 2014, Bakker et al. 2017, Hall et al. 2021). Unfortunately, making older condors – who are more experienced with the landscape, more dominant, and less reliant on food provisioning – more susceptible to lead poisoning compared to younger, recently released birds that depend more on supplementary food at managed release sites (Hall et al. 2007). Furthermore, the large ranging movements of lead-exposed condors – environmental sentinels – highlights the spatially widespread issue of lead contamination across the landscape and is concerning for other avian scavengers in the area.

With regards to our third prediction, regarding how condors share space across the landscape and with each other, we found that clinically affected condors concentrated more within the hunting unit 12 A – a previously identified area of high lead exposure risk (Hunt et al. 2007). Our analysis also clearly showed that lead-exposed individuals overlapped more with each other than with background level birds across the landscape, as we had predicted. However, contrary to expectations, we found that background level birds overlapped more with lead-exposed individuals than with each other, and notably the most with clinically affected individuals. Overlaps of high lead concentration to hunting areas has also been demonstrated in other avian scavengers such as the European griffon vulture (*Gyps fulvus*) (Arrondo et al. 2020).

Together with the range size analyses, these findings indicate that firstly, lead-exposed individuals traverse large areas of the landscape and share space across larger areas. Secondly, they share space with each other more than with individuals who are not exposed to lead. Lastly, background birds move around a smaller area, likely as they are younger individuals depending on supplementary feeding. These background birds are also using these smaller areas in distinct ways, suggestive of potential competition and resource partitioning at predictable supplementary feeding sites (van Overveld et al. 2018). However, it is important to note that our monthly overlaps captured only the joint occurrence of individuals in space and not in time (Benhamou et al. 2014). The lack of observed temporal trends in our analysis highlights the complexity of using GPS-telemetry data for post-exposure detection. Our findings supporting existing literature in that lead-exposed condors cover large areas on both a daily and 30-day overall period, and provides novel insights into how they share space across the landscape.

## Conclusions and future directions

Despite the remarkable comeback from the brink of extinction, lead exposure continues to jeopardize the survival of California Condors across their range. Addressing and detecting lead exposure events are difficult – regulatory changes to limit the sources of environmental lead have proven to be challenging with mixed successes (Schulz et al. 2023); traditional field detection methods, such as blood sampling, are resource-intensive and logistically demanding (Church et al. 2006, Parish et al. 2007); and GPS-telemetry has so far been ineffective in directly detecting changes in movement behavior linked to lead exposure (Poessel et al. 2018), other than in identifying birds that are stationary for long periods (i.e., indicative of potential debiliating health conditions or death) (Cogan et al. 2012). Nonetheless, GPS-telemetry is a useful tool in understanding condors’ spatial patterns in relation to lead exposure. Future research exploring how movement patterns can be used to detect and provide early warnings of at-risk condors may better inform targeted conservation efforts. For instance, in the evaluation of movement metrics in predicting exposure risk or monitoring the development of displacements from supplementary feeding sites or understanding patterns of revisitations and time spent within high-risk zones. Research focused on a proactive approach – applicable to other systems with lead-exposure risks for wildlife – could enable timely interventions, potentially preventing fatal lead poisoning and enhancing the overall effectiveness of conservation efforts. Leveraging GPS telemetry in this way could be a game-changer in the fight to protect and preserve this critically endangered species, and more broadly, species affected by human-induced environmental toxins.

## Supplementary information

Supplementary information

## Data Availability

The dataset of the processed movement metrics and computed overlaps, and associated code, are available on Zenodo at 10.5281/zenodo.15688522.
