# Supplementary material for: Tracking solutions to a persistent threat: spatial movement patterns reflect lead exposure in critically endangered California condors
Source: Ecotoxicology. 2025 Jul 5;34(8):1476–88. doi: 10.1007/s10646-025-02921-9 (PMC12476304; doi:10.1007/s10646-025-02921-9)
Supplement: Supplementary file 1 — Supplementary information [file 10646_2025_2921_MOESM1_ESM.docx]

**Supplementary information**

### Title

Tracking solutions to a persistent threat: Spatial movement patterns reflect lead exposure in critically endangered California Condors

### Authors

Varalika Jain^1,2^, Chris McClure^3^, Chris N. Parish^3^, Tim Hauck^3^, Petra Sumasgutner^1,2^

### Affiliations

^1^ Konrad Lorenz Research Center for Behavior and Cognition, Core facility of the University of Vienna, Grünau im Almtal, 4645 Austria

^2^ Department of Behavioral and Cognitive Biology, University of Vienna, Vienna, 1030 Austria

^3^ The Peregrine Fund, 5668 West Flying Hawk Lane, Boise, ID 83709 USA

### Corresponding author

Varalika Jain

Konrad Lorenz Research Center for Behavior and Cognition, core facility of the University of Vienna, Grünau im Almtal, 4645 Austria

E-mail: [varalika.jain@univie.ac.at](mailto:varalika.jain@univie.ac.at) | Telephone: +4367761066253

(A) (B)


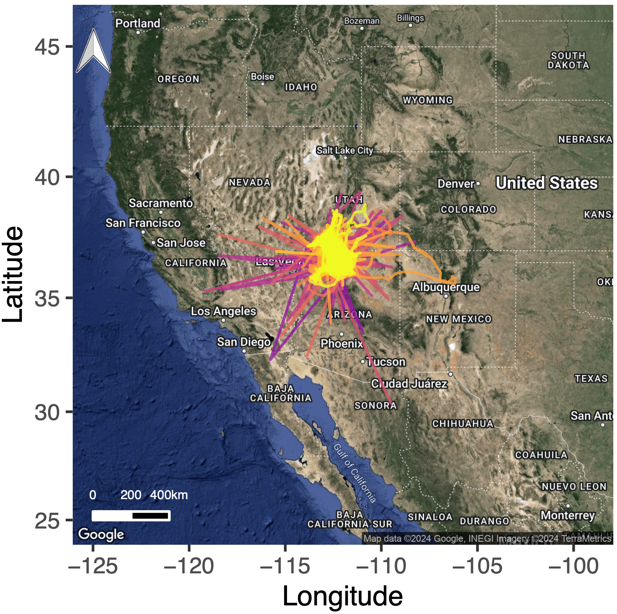

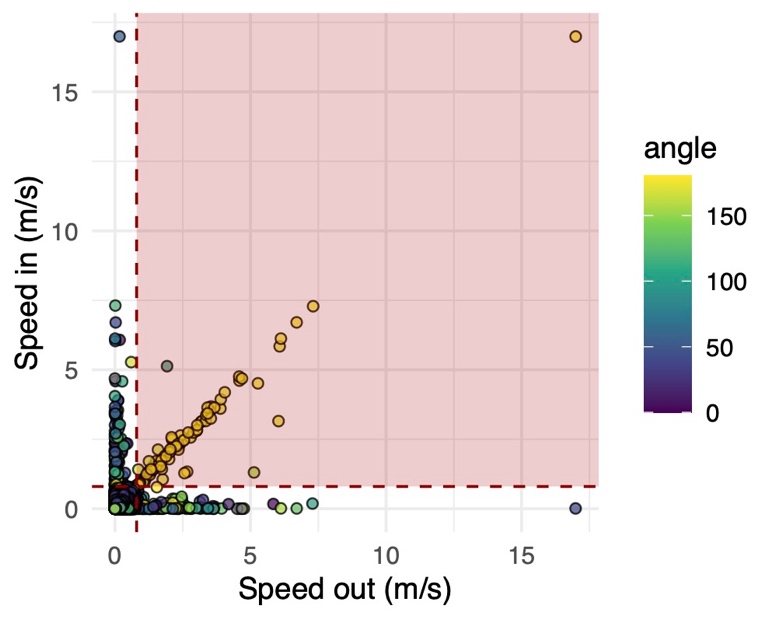


(C)


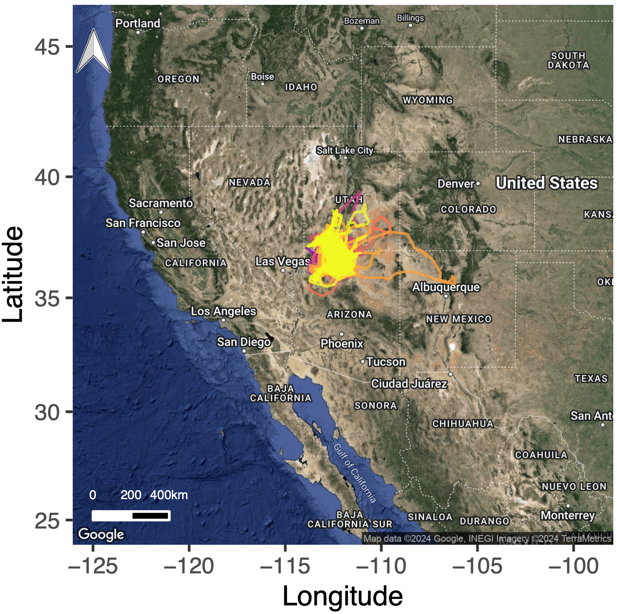


**Figure S1** California Condors were equipped with Microwave Telemetry, Inc. Solar Argos/GPS 50g Patagial PTTs with transmission settings at hourly intervals. We downloaded data from the 14^th^ of January 2015 till the 29^th^ of January 2023. To treat the ‘spikes’ in the data in (A), we first cleaned the data for any outliers based on spatial limits (longitude range: -120 to -90, latitude range: 30 to 39.5). (B) We then calculated angles and speeds in the trajectories, removing points of both high speed (≥ 0.8ms-1, red dashed lines) and large turning angles (≥150 degrees, light green to yellow points) as it would have been unrealistic for the birds to make such sharp turns at high speed (red tinted area). (C) The resulting movements when we applied the angle and speed filter to the data.

**Figure S2** Movement tracks of GPS tagged California Condors of differing lead exposure levels, 30-days prior to when they were tested for lead poisoning.


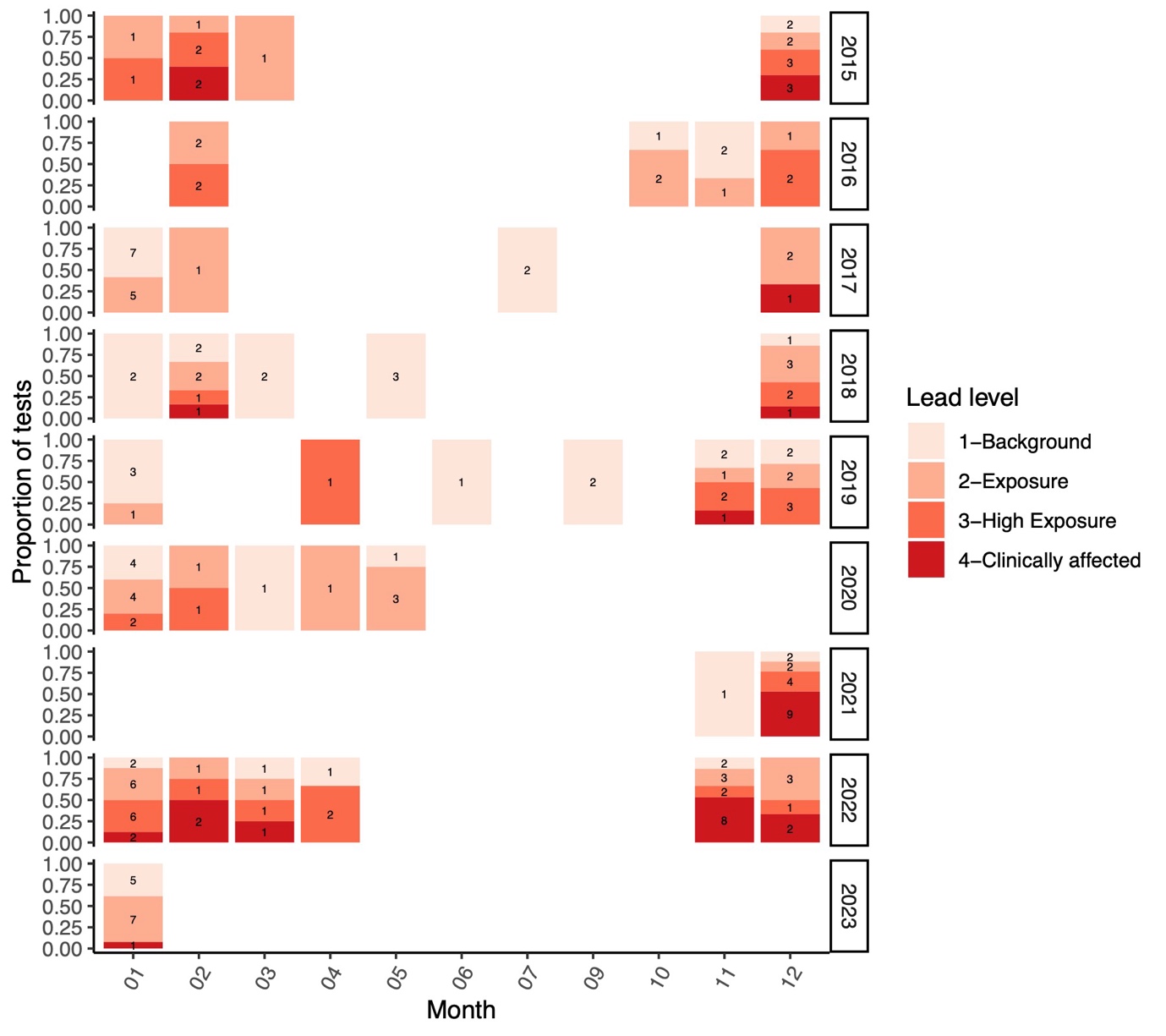


**Figure S3** The proportion of tests revealing California Condors of different lead exposure levels per month, from the 14^th^ of January 2015 till the 29^th^ of January 2023. Testing is often concentrated around hunting season, towards the end and beginning of the year, as condors are more vulnerable to lead contamination during that time. Text in each proportional segment represents the number of tests.

**Table S1** Results of the full model addressing the interaction between lead exposure level and day prior on daily minimum convex polygon (MCP) sizes of California Condors (estimates, together with standard errors, confidence limits, significance tests, as well as minimum and maximum of model estimates obtained after excluding levels of random effects one at a time). Confidence limits were obtained through bootstrapping the data 1000 times and determining the 95% confidence interval.

| **Term** | **Estimate** | **SE** | **lower CI** | **upper CI** | **Chisq** | **df** | **p** | **min** | **max** |
| --- | --- | --- | --- | --- | --- | --- | --- | --- | --- |
| Intercept | 14.228 | 0.438 | 12.991 | 15.427 |  |  |  | 13.954 | 15.065 |
| Exposure^†^ | 0.055 | 0.485 | -1.392 | 1.576 |  |  |  | -0.596 | 0.323 |
| High exposure^†^ | 0.596 | 0.524 | -0.973 | 2.272 |  |  |  | -0.486 | 0.740 |
| Clinically affected^†^ | 1.146 | 0.482 | -0.388 | 2.619 |  |  |  | 0.302 | 1.423 |
| Day prior^‡^ | 0.141 | 0.181 | -0.581 | 0.882 |  |  |  | 0.036 | 0.187 |
| Age^‡^ | 0.534 | 0.268 | -0.253 | 1.303 | 2.225 | 1 | 0.136 | 0.290 | 0.737 |
| Day prior^‡^: exposure^†^ | -0.142 | 0.250 | -1.211 | 0.845 | 1.456 | 3 | 0.693 | -0.232 | -0.045 |
| Day prior^‡^: high exposure^†^ | -0.111 | 0.266 | -1.284 | 0.976 |  |  |  | -0.168 | 0.118 |
| Day prior^‡^: critically affected^†^ | 0.170 | 0.257 | -0.979 | 1.293 |  |  |  | 0.102 | 0.324 |

*‡ = z-transformed to a mean of zero and a standard deviation of one; mean and sd of the original age were 3720.65 and 1887.36 days and for day prior were 15.46 and 8.64 days respectively*

*† = lead exposure level was dummy coded with 'background' being the reference category; the indicated test refers to the overall effect of the interaction*

**Table S2** Results of the full model (Table S1) with regard to the random effects (estimated standard deviation)

| **Grouping variable** | **Effect^*^** | **sd** |
| --- | --- | --- |
| Test nested in individual | Intercept | 1.192 |
| “ | Day prior | 0.760 |
| “ | Age | 0.976 |
| Individual identity | Intercept | 1.079 |
| “ | Exposure | 1.536 |
| “ | High exposure | 1.634 |
| “ | Clinically affected | 1.256 |
| “ | Day prior | 0.271 |
| “ | Age | 0.787 |
| “ | Day prior: exposure | 0.720 |
| “ | Day prior: high exposure | 0.347 |
| “ | Day prior: critically affected | 0.352 |

** 'intercept' depicts to a random intercept, all others to a random slope*

**Table S3** Results of the reduced model (lacking the interaction between lead level and day prior; Table S1) on daily minimum convex polygon (MCP) sizes

| **Term** | **Estimate** | **SE** | **Chisq** | **df** | **p** |
| --- | --- | --- | --- | --- | --- |
| Intercept | 14.254 | 0.410 |  |  |  |
| Exposure^†^ | 0.080 | 0.478 | 61.884 | 3 | 0.000 |
| High exposure^†^ | 0.608 | 0.505 |  |  |  |
| Clinically affected^†^ | 1.151 | 0.472 |  |  |  |
| Day prior^‡^ | 0.116 | 0.096 | 0.238 | 1 | 0.626 |
| Age^‡^ | 0.524 | 0.261 | 2.107 | 1 | 0.147 |

*‡ = z-transformed to a mean of zero and a standard deviation of one; mean and sd of the original age were 3720.65 and 1887.36 days and for day prior were 15.46 and 8.64 days respectively*

*† = lead exposure level was dummy coded with 'Background' being the reference category; the indicated test refers to the overall effect of the interaction*

**Table S4** Results of the pairwise comparison of lead exposure levels from Table S3. The numbers depicted in bold show significant results, the numbers depicted in italics show trends

| **Reference level** | **Comparison with** | **Estimate** | **SE** | **z-ratio** | **p** |
| --- | --- | --- | --- | --- | --- |
| Background | Exposure | -0.080 | 0.478 | -0.168 | 0.998 |
| Background | High Exposure | -0.608 | 0.505 | -1.204 | 0.624 |
| *Background* | *Clinically affected* | *-1.151* | *0.472* | *-2.441* | *0.070* |
| Exposure | High Exposure | -0.527 | 0.400 | -1.318 | 0.551 |
| **Exposure** | **Clinically affected** | **-1.070** | **0.395** | **-2.710** | **0.034** |
| High Exposure | Clinically affected | -0.543 | 0.417 | -1.304 | 0.561 |

**Table S5** Results of the full model addressing the proportion of overlap between California Condors of different lead exposure level combinations (estimates, together with standard errors, confidence limits, significance tests, as well as minimum and maximum of model estimates obtained after excluding levels of random effects one at a time). Confidence limits were obtained through bootstrapping the data 100 times and determining the 95% confidence interval.

| **Term** | **Estimate** | **SE** | **lower CI** | **upper CI** | **Chisq** | **df** | **p** | **min** | **max** |
| --- | --- | --- | --- | --- | --- | --- | --- | --- | --- |
| (Intercept) | 0.093 | 0.032 | 0.044 | 0.141 |  |  |  | 0.090 | 0.099 |
| Background-Exposure^†^ | 0.237 | 0.043 | 0.118 | 0.264 | 613.857 | 15 | <0.001 | 0.230 | 0.243 |
| Background-High exposure^†^ | 0.263 | 0.051 | 0.139 | 0.298 |  |  |  | 0.256 | 0.268 |
| Background-Clinically affected^†^ | 0.377 | 0.052 | 0.237 | 0.400 |  |  |  | 0.369 | 0.385 |
| Exposure-Background^†^ | -0.397 | 0.041 | -0.428 | -0.293 |  |  |  | -0.403 | -0.393 |
| Exposure-Exposure^†^ | -0.058 | 0.039 | -0.129 | 0.013 |  |  |  | -0.066 | -0.052 |
| Exposure-High exposure^†^ | -0.020 | 0.047 | -0.110 | 0.048 |  |  |  | -0.026 | -0.015 |
| Exposure-Clinically affected^†^ | 0.099 | 0.048 | 0.011 | 0.161 |  |  |  | 0.094 | 0.104 |
| High Exposure-Background^†^ | -0.415 | 0.045 | -0.443 | -0.308 |  |  |  | -0.420 | -0.411 |
| High Exposure-Exposure^†^ | -0.092 | 0.046 | -0.159 | 0.002 |  |  |  | -0.097 | -0.087 |
| High Exposure-High exposure^†^ | -0.073 | 0.049 | -0.164 | 0.009 |  |  |  | -0.078 | -0.064 |
| High Exposure-Clinically affected^†^ | 0.090 | 0.053 | -0.004 | 0.171 |  |  |  | 0.082 | 0.096 |
| Clinically affected-Background^†^ | -0.471 | 0.046 | -0.487 | -0.344 |  |  |  | -0.477 | -0.464 |
| Clinically affected-Exposure^†^ | -0.136 | 0.046 | -0.194 | -0.059 |  |  |  | -0.143 | -0.131 |
| Clinically affected-High exposure^†^ | -0.092 | 0.053 | -0.162 | 0.002 |  |  |  | -0.100 | -0.087 |
| Clinically affected-Clinically affected^†^ | 0.019 | 0.053 | -0.062 | 0.123 |  |  |  | 0.009 | 0.027 |

*†= lead level combination was dummy coded with 'background-background' being the reference category; the indicated test refers to the overall effect of the interaction*

**Table S6** Results of the full model (Table S5) with regard to the random effects (estimated standard deviation)

| **Grouping variable** | **Effect*** | **sd** |
| --- | --- | --- |
| Individual identity dyad | (Intercept) | 0.432 |
|  | Background-Exposure | 0.570 |
|  | Background-High exposure | 0.682 |
|  | Background-Clinically affected | 0.710 |
|  | Exposure-Background | 0.427 |
|  | Exposure-Exposure | 0.102 |
|  | Exposure-High exposure | 0.505 |
|  | Exposure-Clinically affected | 0.476 |
|  | High Exposure-Background | 0.362 |
|  | High Exposure-Exposure | 0.392 |
|  | High Exposure-High exposure | 0.000 |
|  | High Exposure-Clinically affected | 0.423 |
|  | Clinically affected-Background | 0.352 |
|  | Clinically affected-Exposure | 0.315 |
|  | Clinically affected-High exposure | 0.388 |
|  | Clinically affected-Clinically affected | 0.000 |

** 'intercept' depicts to a random intercept, all others to a random slope*

**Table S7** Results of the pairwise comparison of lead exposure levels from Table S5. The numbers depicted in bold show significant results, the numbers depicted in italics show trends

| **Reference level** | **Comparison with** | **Estimate** | **SE** | **z-value** | **p** |
| --- | --- | --- | --- | --- | --- |
| **Background-Background** | **Background-Exposure** | **-0.237** | **0.043** | **-5.456** | **<0.001** |
| **Background-Background** | **Background-High exposure** | **-0.263** | **0.051** | **-5.207** | **<0.001** |
| **Background-Background** | **Background-Clinically affected** | **-0.377** | **0.053** | **-7.186** | **<0.001** |
| **Background-Background** | **Exposure-Background** | **0.397** | **0.041** | **9.686** | **<0.001** |
| Background-Background | Exposure-Exposure | 0.058 | 0.039 | 1.487 | 0.984 |
| Background-Background | Exposure-High exposure | 0.020 | 0.047 | 0.422 | 1.000 |
| Background-Background | Exposure-Clinically affected | -0.100 | 0.048 | -2.084 | 0.776 |
| **Background-Background** | **High exposure-Background** | **0.415** | **0.045** | **9.212** | **<0.001** |
| Background-Background | High exposure-Exposure | 0.092 | 0.046 | 2.012 | 0.819 |
| Background-Background | High exposure-High exposure | 0.073 | 0.049 | 1.476 | 0.985 |
| Background-Background | High exposure-Clinically affected | -0.090 | 0.053 | -1.696 | 0.948 |
| **Background-Background** | **Clinically affected-Background** | **0.471** | **0.046** | **10.181** | **<0.001** |
| Background-Background | Clinically affected-Exposure | 0.136 | 0.046 | 2.958 | 0.187 |
| Background-Background | Clinically affected-High exposure | 0.092 | 0.053 | 1.732 | 0.939 |
| Background-Background | Clinically affected-Clinically affected | -0.019 | 0.053 | -0.365 | 1.000 |
| Background-Exposure | Background-High exposure | -0.027 | 0.053 | -0.498 | 1.000 |
| Background-Exposure | Background-Clinically affected | -0.140 | 0.055 | -2.554 | 0.431 |
| **Background-Exposure** | **Exposure-Background** | **0.634** | **0.044** | **14.400** | **<0.001** |
| **Background-Exposure** | **Exposure-Exposure** | **0.295** | **0.041** | **7.126** | **<0.001** |
| **Background-Exposure** | **Exposure-High exposure** | **0.257** | **0.049** | **5.220** | **<0.001** |
| Background-Exposure | Exposure-Clinically affected | 0.137 | 0.050 | 2.757 | 0.295 |
| **Background-Exposure** | **High exposure-Background** | **0.652** | **0.048** | **13.493** | **<0.001** |
| **Background-Exposure** | **High exposure-Exposure** | **0.329** | **0.048** | **6.867** | **<0.001** |
| **Background-Exposure** | **High exposure-High exposure** | **0.310** | **0.052** | **5.997** | **<0.001** |
| Background-Exposure | High exposure-Clinically affected | 0.147 | 0.055 | 2.659 | 0.358 |
| **Background-Exposure** | **Clinically affected-Background** | **0.708** | **0.049** | **14.396** | **<0.001** |
| **Background-Exposure** | **Clinically affected-Exposure** | **0.373** | **0.048** | **7.745** | **<0.001** |
| **Background-Exposure** | **Clinically affected-High exposure** | **0.328** | **0.055** | **5.979** | **<0.001** |
| Background-Exposure | Clinically affected-Clinically affected | 0.217 | 0.055 | 3.969 | 0.007 |
| Background-High exposure | Background-Clinically affected | -0.114 | 0.061 | -1.870 | 0.890 |
| **Background-High exposure** | **Exposure-Background** | **0.661** | **0.052** | **12.802** | **<0.001** |
| **Background-High exposure** | **Exposure-Exposure** | **0.322** | **0.049** | **6.524** | **<0.001** |
| **Background-High exposure** | **Exposure-High exposure** | **0.283** | **0.055** | **5.129** | **<0.001** |
| Background-High exposure | Exposure-Clinically affected | 0.164 | 0.056 | 2.910 | 0.210 |
| **Background-High exposure** | **High exposure-Background** | **0.678** | **0.054** | **12.642** | **<0.001** |
| **Background-High exposure** | **High exposure-Exposure** | **0.355** | **0.054** | **6.563** | **<0.001** |
| **Background-High exposure** | **High exposure-High exposure** | **0.336** | **0.057** | **5.901** | **<0.001** |
| Background-High exposure | High exposure-Clinically affected | 0.173 | 0.061 | 2.858 | 0.237 |
| **Background-High exposure** | **Clinically affected-Background** | **0.734** | **0.056** | **13.190** | **<0.001** |
| **Background-High exposure** | **Clinically affected-Exposure** | **0.400** | **0.055** | **7.274** | **<0.001** |
| **Background-High exposure** | **Clinically affected-High exposure** | **0.355** | **0.060** | **5.878** | **<0.001** |
| **Background-High exposure** | **Clinically affected-Clinically affected** | **0.244** | **0.061** | **4.023** | **0.006** |
| **Background-Clinically affected** | **Exposure-Background** | **0.774** | **0.053** | **14.555** | **<0.001** |
| **Background-Clinically affected** | **Exposure-Exposure** | **0.435** | **0.051** | **8.547** | **<0.001** |
| **Background-Clinically affected** | **Exposure-High exposure** | **0.397** | **0.057** | **6.934** | **<0.001** |
| **Background-Clinically affected** | **Exposure-Clinically affected** | **0.278** | **0.057** | **4.837** | **<0.001** |
| **Background-Clinically affected** | **High exposure-Background** | **0.792** | **0.056** | **14.050** | **<0.001** |
| **Background-Clinically affected** | **High exposure-Exposure** | **0.469** | **0.056** | **8.349** | **<0.001** |
| **Background-Clinically affected** | **High exposure-High exposure** | **0.450** | **0.059** | **7.591** | **<0.001** |
| **Background-Clinically affected** | **High exposure-Clinically affected** | **0.287** | **0.062** | **4.624** | **<0.001** |
| **Background-Clinically affected** | **Clinically affected-Background** | **0.848** | **0.056** | **15.083** | **<0.001** |
| **Background-Clinically affected** | **Clinically affected-Exposure** | **0.513** | **0.056** | **9.161** | **<0.001** |
| **Background-Clinically affected** | **Clinically affected-High exposure** | **0.469** | **0.062** | **7.575** | **<0.001** |
| **Background-Clinically affected** | **Clinically affected-Clinically affected** | **0.358** | **0.061** | **5.823** | **<0.001** |
| **Exposure-Background** | **Exposure-Exposure** | **-0.339** | **0.039** | **-8.682** | **<0.001** |
| **Exposure-Background** | **Exposure-High exposure** | **-0.377** | **0.047** | **-7.988** | **<0.001** |
| **Exposure-Background** | **Exposure-Clinically affected** | **-0.497** | **0.048** | **-10.353** | **<0.001** |
| Exposure-Background | High exposure-Background | 0.018 | 0.046 | 0.382 | 1.000 |
| **Exposure-Background** | **High exposure-Exposure** | **-0.306** | **0.046** | **-6.674** | **<0.001** |
| **Exposure-Background** | **High exposure-High exposure** | **-0.324** | **0.050** | **-6.508** | **<0.001** |
| **Exposure-Background** | **High exposure-Clinically affected** | **-0.487** | **0.054** | **-9.115** | **<0.001** |
| Exposure-Background | Clinically affected-Background | 0.073 | 0.047 | 1.561 | 0.975 |
| **Exposure-Background** | **Clinically affected-Exposure** | **-0.261** | **0.046** | **-5.656** | **<0.001** |
| **Exposure-Background** | **Clinically affected-High exposure** | **-0.306** | **0.053** | **-5.742** | **<0.001** |
| **Exposure-Background** | **Clinically affected-Clinically affected** | **-0.417** | **0.053** | **-7.841** | **<0.001** |
| Exposure-Exposure | Exposure-High exposure | -0.038 | 0.044 | -0.878 | 1.000 |
| **Exposure-Exposure** | **Exposure-Clinically affected** | **-0.158** | **0.044** | **-3.575** | **0.031** |
| **Exposure-Exposure** | **High exposure-Background** | **0.357** | **0.044** | **8.158** | **<0.001** |
| Exposure-Exposure | High exposure-Exposure | 0.034 | 0.042 | 0.799 | 1.000 |
| Exposure-Exposure | High exposure-High exposure | 0.015 | 0.047 | 0.320 | 1.000 |
| Exposure-Exposure | High exposure-Clinically affected | -0.148 | 0.050 | -2.940 | 0.196 |
| **Exposure-Exposure** | **Clinically affected-Background** | **0.413** | **0.045** | **9.236** | **<0.001** |
| Exposure-Exposure | Clinically affected-Exposure | 0.078 | 0.042 | 1.854 | 0.896 |
| Exposure-Exposure | Clinically affected-High exposure | 0.034 | 0.050 | 0.669 | 1.000 |
| Exposure-Exposure | Clinically affected-Clinically affected | -0.077 | 0.050 | -1.552 | 0.976 |
| Exposure-High exposure | Exposure-Clinically affected | -0.119 | 0.052 | -2.306 | 0.619 |
| **Exposure-High exposure** | **High exposure-Background** | **0.395** | **0.050** | **7.851** | **<0.001** |
| Exposure-High exposure | High exposure-Exposure | 0.072 | 0.049 | 1.475 | 0.985 |
| Exposure-High exposure | High exposure-High exposure | 0.053 | 0.052 | 1.019 | 1.000 |
| Exposure-High exposure | High exposure-Clinically affected | -0.110 | 0.056 | -1.954 | 0.850 |
| **Exposure-High exposure** | **Clinically affected-Background** | **0.451** | **0.052** | **8.705** | **<0.001** |
| Exposure-High exposure | Clinically affected-Exposure | 0.116 | 0.050 | 2.323 | 0.607 |
| Exposure-High exposure | Clinically affected-High exposure | 0.072 | 0.056 | 1.284 | 0.997 |
| Exposure-High exposure | Clinically affected-Clinically affected | -0.039 | 0.057 | -0.693 | 1.000 |
| **Exposure-Clinically affected** | **High exposure-Background** | **0.514** | **0.052** | **9.966** | **<0.001** |
| **Exposure-Clinically affected** | **High exposure-Exposure** | **0.191** | **0.050** | **3.790** | **0.014** |
| Exposure-Clinically affected | High exposure-High exposure | 0.172 | 0.054 | 3.188 | 0.102 |
| Exposure-Clinically affected | High exposure-Clinically affected | 0.009 | 0.057 | 0.164 | 1.000 |
| **Exposure-Clinically affected** | **Clinically affected-Background** | **0.570** | **0.052** | **10.981** | **<0.001** |
| **Exposure-Clinically affected** | **Clinically affected-Exposure** | **0.236** | **0.050** | **4.755** | **<0.001** |
| *Exposure-Clinically affected* | *Clinically affected-High exposure* | *0.191* | *0.057* | *3.373* | *0.059* |
| Exposure-Clinically affected | Clinically affected-Clinically affected | 0.080 | 0.056 | 1.438 | 0.989 |
| **High exposure-Background** | **High exposure-Exposure** | **-0.323** | **0.049** | **-6.603** | **<0.001** |
| **High exposure-Background** | **High exposure-High exposure** | **-0.342** | **0.052** | **-6.556** | **<0.001** |
| **High exposure-Background** | **High exposure-Clinically affected** | **-0.505** | **0.056** | **-8.992** | **<0.001** |
| High exposure-Background | Clinically affected-Background | 0.056 | 0.051 | 1.105 | 0.999 |
| **High exposure-Background** | **Clinically affected-Exposure** | **-0.279** | **0.050** | **-5.583** | **<0.001** |
| **High exposure-Background** | **Clinically affected-High exposure** | **-0.323** | **0.056** | **-5.784** | **<0.001** |
| **High exposure-Background** | **Clinically affected-Clinically affected** | **-0.434** | **0.056** | **-7.721** | **<0.001** |
| High exposure-Exposure | High exposure-High exposure | -0.019 | 0.051 | -0.367 | 1.000 |
| *High exposure-Exposure* | *High exposure-Clinically affected* | *-0.182* | *0.055* | *-3.301* | *0.074* |
| **High exposure-Exposure** | **Clinically affected-Background** | **0.379** | **0.051** | **7.513** | **<0.001** |
| High exposure-Exposure | Clinically affected-Exposure | 0.045 | 0.049 | 0.914 | 1.000 |
| High exposure-Exposure | Clinically affected-High exposure | 0.000 | 0.055 | 0.000 | 1.000 |
| High exposure-Exposure | Clinically affected-Clinically affected | -0.111 | 0.055 | -2.007 | 0.822 |
| High exposure-High exposure | High exposure-Clinically affected | -0.163 | 0.058 | -2.830 | 0.252 |
| **High exposure-High exposure** | **Clinically affected-Background** | **0.398** | **0.054** | **7.366** | **<0.001** |
| High exposure-High exposure | Clinically affected-Exposure | 0.063 | 0.053 | 1.202 | 0.998 |
| High exposure-High exposure | Clinically affected-High exposure | 0.019 | 0.057 | 0.326 | 1.000 |
| High exposure-High exposure | Clinically affected-Clinically affected | -0.092 | 0.058 | -1.580 | 0.972 |
| **High exposure-Clinically affected** | **Clinically affected-Background** | **0.561** | **0.057** | **9.843** | **<0.001** |
| **High exposure-Clinically affected** | **Clinically affected-Exposure** | **0.226** | **0.055** | **4.084** | **0.005** |
| High exposure-Clinically affected | Clinically affected-High exposure | 0.182 | 0.060 | 3.024 | 0.159 |
| High exposure-Clinically affected | Clinically affected-Clinically affected | 0.071 | 0.061 | 1.170 | 0.999 |
| **Clinically affected-Background** | **Clinically affected-Exposure** | **-0.334** | **0.050** | **-6.657** | **<0.001** |
| **Clinically affected-Background** | **Clinically affected-High exposure** | **-0.379** | **0.057** | **-6.679** | **<0.001** |
| **Clinically affected-Background** | **Clinically affected-Clinically affected** | **-0.490** | **0.056** | **-8.707** | **<0.001** |
| Clinically affected-Exposure | Clinically affected-High exposure | -0.045 | 0.055 | -0.807 | 1.000 |
| Clinically affected-Exposure | Clinically affected-Clinically affected | -0.156 | 0.054 | -2.871 | 0.230 |
| Clinically affected-High exposure | Clinically affected-Clinically affected | -0.111 | 0.060 | -1.840 | 0.902 |
